# Specific Recognition of Arginine Methylated Histone Tails by JMJD5 and JMJD7

**DOI:** 10.1038/s41598-018-21432-8

**Published:** 2018-02-19

**Authors:** Haolin Liu, Chao Wang, Schuyler Lee, Fangkun Ning, Yang Wang, Qianqian Zhang, Zhongzhou Chen, Jianye Zang, Jay Nix, Shaodong Dai, Philippa Marrack, James Hagman, John Kappler, Gongyi Zhang

**Affiliations:** 1 Department of Biomedical Research, National Jewish Health, 1400 Jackson St, Denver, CO 80206 USA; 2 State Key Laboratory of Agrobiotechnology, Chinese Agricultural University, Beijing, 100193 P. R. China; 3 Department of Molecular Biology, University of Science and Technology of China, Hefei, 900015 P. R. China; 4 Molecular Biology Consortium, Advanced Light Source, Lawrence Berkeley National Laboratory, Berkeley, California, 94720 USA; 5 Department of Immunology and Microbiology, School of Medicine, University of Colorado Denver, Denver, CO 80206 USA; 6 Howard Hughes Medical Institute, Denver, CO 80206 USA

## Abstract

We have reported that JMJD5 and JMJD7 (JMJD5/7) are responsible for the clipping of arginine methylated histone tails to generate “tailless nucleosomes”, which could release the pausing RNA polymerase II (Pol II) into productive transcription elongation. JMJD5/7 function as endopeptidases that cleave histone tails specifically adjacent to methylated arginine residues and continue to degrade N-terminal residues of histones via their aminopeptidase activity. Here, we report structural and biochemical studies on JMJD5/7 to understand the basis of substrate recognition and catalysis mechanism by this JmjC subfamily. Recognition between these enzymes and histone substrates is specific, which is reflected by the binding data between enzymes and substrates. High structural similarity between JMJD5 and JMJD7 is reflected by the shared common substrates and high binding affinity. However, JMJD5 does not bind to arginine methylated histone tails with additional lysine acetylation while JMJD7 does not bind to arginine methylated histone tails with additional lysine methylation. Furthermore, the complex structures of JMJD5 and arginine derivatives revealed a Tudor domain-like binding pocket to accommodate the methylated sidechain of arginine, but not lysine. There also exists a glutamine close to the catalytic center, which may suggest a unique imidic acid mediated catalytic mechanism for proteolysis by JMJD5/7.

## Introduction

The phenomenon of cleavage at histone tails has been revealed several decades ago^1^, which is consistent with reports of high turnover rate of histone subunits within non-proliferating cells^2^. Similarly, arginine methylation on histone tails is very well characterized and a family containing at least 9 arginine methyltransferases has been established^3^. The reversibility hallmark of epigenetics indicates there must exist an enzyme family responsible for the removal of methyl groups on arginines. To this regard, JMJD6 was first reported to have such ability by two groups^4,5^. Interestingly, another group claimed that lysine demethylases also contain arginine demethylase activities^6^. On the other hand, it remains a mystery as to how phosphorylation of CTD of RNA Polymerase II (Pol II) by CDK9 release the pausing Pol II into productive elongation. It was reported that nucleosomes at +1 position is the major barrier to block the Pol II from elongation^7^. It is still unknown how nucleosomes at +1 are removed during transcription elongation. Our recent discovery revealed that JMJD5 and JMJD7 specifically make first cleavage at methylarginine sites on histone tails through endopeptidase activities and continue to trim histone tails with aminopeptidase activities to generate “tailless nucleosomes”^8^, which should be overcome by Pol II without further additional assistance, just as that of Archaea bacteria^9^. We also found that the cleavage activities of both JMJD5 and JMJD7 are essential for proliferation of mice fibroblast cells and breast cancer cells respectively^8^. Furthermore, both arginine methylated histones and overall histone content are dramatically increased in both JMJD5 and JMJD7 deficient cells, suggesting that both JMJD5 and JMJD7 regulate the homeostasis of histones^8^. Our data suggest that clipping of histone tails, “tailless nucleosomes” generation, arginine methylation, high turnover rates of histone, and Pol II elongation are intricately linked. Together, they suggest a general transcription mechanism for stimulating genes controlled by Pol II pausing, which are reported to represent a high percentage of all genes (~30–90%) in mice and human^10,11^. However, a big mystery raised from this research is how a subfamily of Jumonji C (JmjC) domain containing hydroxylases is adapted to function as a protease family.

JmjC domain containing protein family includes over 60 members, many of which carry out hydroxylation of small molecules, proteins, and nucleotides^12,13^. Many JmjC family members have demethylase activity, including removal of methyl groups from methylated lysine residues of histone tails^14,15^, methylated bases or other sites on RNA^5,16,17^, and methylated 5′-cytosine or other sites on DNA^12,18^, thus settling their status as key players in epigenetic regulation of gene transcription.

The JmjC protein JMJD5 was first reported to remove methyl groups from H3K36 and plays critical roles in cell cycle regulation, embryonic development, and cancer cell proliferation^19–23^. However, the lysine demethylase activity of JMJD5 was not reproduced in another report^24^; these and other analyses detected unique structural features of JMJD5 suggesting substrates other than methylated lysine^24,25^ (RCSB ID:3UYJ and 4AAP). During our pursuit in the characterization of potential histone arginine demethylases, we found that the catalytic cores of both JMJD5 and JMJD7 specifically cleave arginine methylated histone tails through both endopeptidase and exopeptidase activities^8^. The question arises, how does a putative hydroxylase generate protease activity? What are the structural basis and the specific recognition mode by which these enzymes recognize their substrates? What is the mechanism of catalysis? How do JMJD5 and JMJD7 differentiate their own substrates? To address these questions, we have determined a series of structures of c-JMJD5, JMJD7, and c-JMJD5 with substrates, coupled with biochemical data.

### The structure of JMJD7

**Figure 1:**
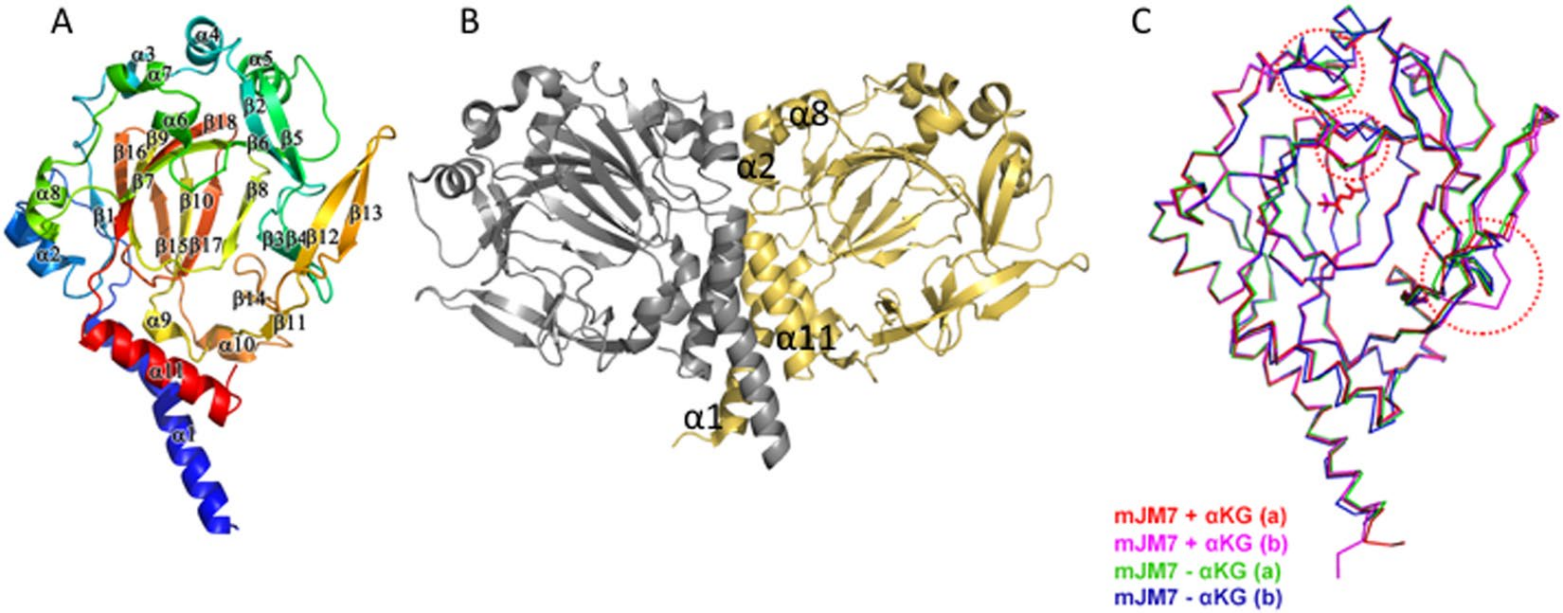
Structures of JMJD7. (**A**) A monomer structure of JMJD7 (4QSZ). (**B**) The potential biological dimer (4QSZ). (**C**) Comparison of individual JMJD7 from different crystal packing structures (4QSZ, red and purple; 4QU2, green and blue). Dotted red circles indicate regions with differences between JMJD7 structures.

We crystallized multiple versions of mouse JMJD7, including a MBP-JMJD7 fusion protein (with a truncated linker) and a JMJD7 protein alone (generated from GST-JMJD7 fusion protein). The structure of MBP-JMJD7 was determined using the molecular replacement method with both MBP (PDB:1HSJ)^26^ and c-JMJD5 (PDB: 4QU1) as starting models (Table S1, Table S2, Table S3). Two molecules of MBP-JMJD7, which form a dimer through two helix bundles (each one is formed by one helix from the N-terminus and another one from the C-terminus), assembled one asymmetric unit with slightly different conformations in the absence of α-KG (Fig. 1, Fig. S1). The structure of the JMJD7 alone version was determined by molecular replacement of the JMJD7 model from the MBP-JMJD7 structure in the presence of α-KG. Again, two molecules of JMJD7, which formed dimers similar to those of the MBP-JMJD7 fusion (suggesting that the dimer is not an artificial one caused by MBP or crystal packing), were observed in one asymmetric unit with slightly different conformations (Fig. 1, Fig. S2). Besides the two helix bundles, additional helices participated in protein-protein interactions (Fig. 1B). This dimeric form may represent a functionally relevant form of the protein *in vivo*.

**Figure 2:**
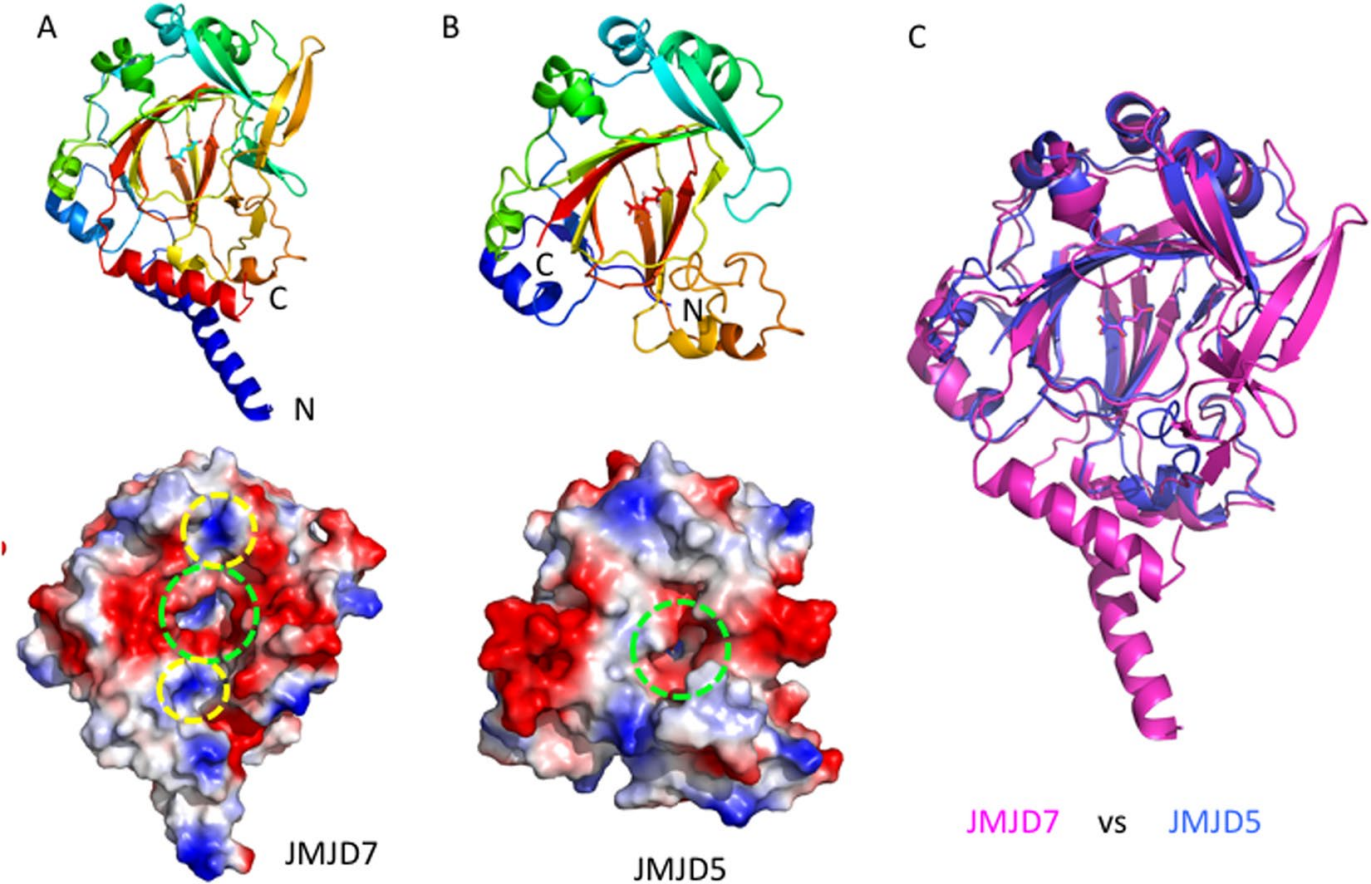
Comparisons of JMJD5 and JMJD7. (**A**) Structure and surface charge distributions of JMJD7. (**B**) Structure and surface charge distribution of JMJD5. All surface charges figures in the context were generated using PyMOL (Action > generate > vacuum electrostatics > protein contact potential) (https://pymol.org/2/). Red represents negatively-charged surface, Gray represents neutral-charged surface, and Blue represents positively-charged surface (contour level from negative charge −49.921 to positive charge + 49.921). Dotted green circle indicates the active site. Dotted yellow circle indicates uniquely positively-charged patches near JMJD7 active site. (**C**) The overlap of JMJD5 on JMJD7. Top- ribbon model. Bottom- surface charges.

Comparing all four JMJD7 structures (two MBP-JMJD7 fusions, two GST-free JMJD7, each with or without α-KG), we found several interesting differences (Fig. 1C). First, marginal conformational changes were detected in the active site with and without α-KG (Fig. 1 and Fig. S2). Second, discrete regions were flexible and adopted variable conformations in different packing environments (red dotted circles in Fig. 1C and difference in surface charge distribution in Fig. S2). Similar to all JmjC domain containing proteins, JMJD7 has the Cupin signature fold at the catalytic core including the Fe^2+^ and α-KG binding site (Fig. 2A). Several unique structural features are worth noting. First, a helix from the N-terminus (α1, colored blue) and another helix from the C-terminus (α11, colored red) form a coil-coil helical bundle, which participates in dimer formation (Fig. 1A). Second, compared to other JmjC domain containing proteins, two extra beta hairpins generated by β3 and β4 and β12 and β13, cross each other to help form the potential substrate binding site (Fig. 1A, Fig. S3). Furthermore, the unique surface charge distribution of JMJD7 suggests that it may accommodate highly charged histone tails (Fig. 2A). A negatively charged catalytic center is surrounded by nearby distribution of positively charged patches, indicating a preference for positively charged sidechains of methylarginine at the catalytic core, while accommodating neutral or negatively charged neighboring residues on histone tail substrates (Fig. 2A).

### Comparisons of JMJD7 to other JmjC proteins

Numerous structures of JmjC protein family members have been determined (Fig. S4), including the lysine hydroxylase FIH^27^, lysine demethylase JMJD2A^28,29^, RNA demethylases FTO and TYW5^17,30^, DNA demethylase AlkB^31,32^, and DNA 5′-cytosine hydroxylases including Ten Eleven Translocase 2^33^. Sequence alignments reveal how proteins are related evolutionarily, while structural comparisons validate or invalidate the accuracy of the sequence alignment. Our sequence alignment indicates that JMJD7 (residues from 27–294) is highly similar to JMJD5 (residues from 183–416) with 25.1% identity and 43.6% similarity (Fig. S3). Not surprisingly, the structure of JMJD7 overlays well with that of JMJD5 (residues 183–416, c-JMJD5) lacking the N-terminal domain, whose structure has been reported by numerous groups^24,25^ including our own (RSCB ID: 4QU1, Table S1). The RMSD is less than 2.0 Å (230 Cα, excluding two beta hairpins formed by β3 and β4 and β12 and β13) using the Dali program (http://ekhidna.biocenter.helsinki.fi/dali_server/) (Fig. 2C). The main difference between c-JMJD5 and JMJD7 is that JMJD7 has an extra two helix bundle and two extra beta hairpins, both of which may participate in dimer formation (Fig. 2C, Fig. S3). The most striking similarity between these two structures is the surface charge distribution; both contain concentrations of negative charge at the catalytic center (Fig. 2A,B). The structural similarity indicates common functional roles. We propose that both c-JMJD5 and JMJD7 recognize positively charged moieties including methylated arginine residues. However, significant differences in charge distribution near the catalytic center were also revealed; there are two flanking positive patches adjacent to the catalytic center on JMJD7, but not on c-JMJD5 (Fig. 2A,B).

JMJD6 has been reported to possess arginine demethylase activity toward both H3R2 and H4R3^4,5^. Therefore, we examined whether structural similarities could be identified between JMJD6 (residues 120–334) and JMJD7/c-JMJD5. Overall, the differences are significant with RMSD of 3.3 Å (Fig. S4). Interestingly, we found similar negative charge distributions at the proteins’ catalytic centers, but dramatic differences in surrounding residues between JMJD6 and JMJD7/c-JMJD5 (Fig. S4). This analysis suggests that JMJD6 may recognize substrates that are similarly charged at the catalytic center, but have significantly different neighboring charge distributions relative to c-JMJD5 and JMJD7. This result is consistent with our early report that the catalytic core of JMJD6 (residues 1–334) barely binds to single strand RNA (ssRNA) though the entire JMJD6 (1–403) nonspecifically recognizes ssRNA with high binding affinity^16^, suggesting that ssRNAs are not substrates of JMJD6. However, it is possible that binding between JMJD6 and single stranded RNA may recruit JMJD6 to its actual substrate(s).

### The complex structure of c-JMJD5 and a symmetric di-methyl arginine derivative

**Figure 3:**
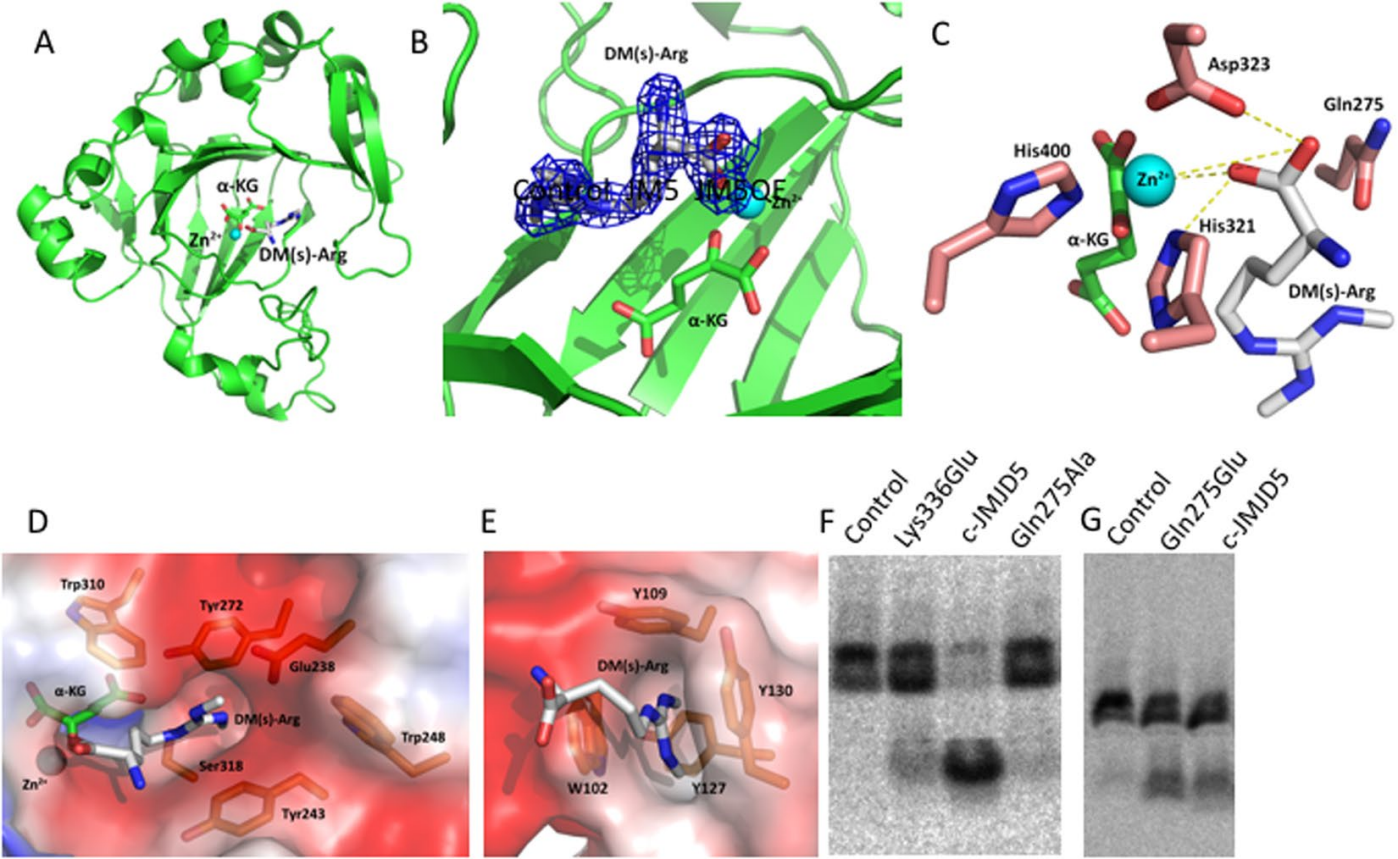
The complex structure of c-JMJD5 and a symmetric dimethyl arginine (DM(s)-Arg). (**A**) Complex structure of DM(s)-Arg and c-JMJD5. (**B**) Omit map 2Fo-Fc electron density of DM(s)-Arg with contour level 1σ. (**C**) The coordination of elements at catalytic center. (**D**) The binding pocket for methylated guadindine group of arginine. DM(s)-Arg, symmetric dimethyl arginine. (**E**) Tudor domain from protein SMN (PDB ID: 4A4E) and asymmetric dimethyl-Arg. (**F**) Proteolytic activities of c-JMJD5 and different mutated versions on radioactively labeled bulk histone. A successful proteolytic activity is indicated by the appearance of a smaller molecular weight product, as seen with c-JMJD5. The activity of c-JMJD5 dropped dramatically after mutation of Lys336 to Glu, which affects α-KG binding. The activity of c-JMJD5 is almost abolished after mutation of Gln275 to Ala, which may affect proton transfer during catalysis. Control refers substrate alone without enzyme. (**G**) A point mutation of Gln275 to Glu275 confers c-JMJD5 a higher enzymatic activity. Substrates are generated as in Fig. 3F.

All JmjC domain containing proteins characterized to date have oxygenase activities^13^. The catalytic cores of both JMJD5 and JMJD7 are structurally similar to other JmjC proteins. What structural features within c-JMJD5 and JMJD7 are responsible for the peptidase activities? As we previously reported, activities of c-JMJD5 and JMJD7 are dependent on divalent cations, similar to typical metalloproteases^8^. Comparison of c-JMJD5 and JMJD7 structures to those of metalloproteases revealed that both c-JMJD5 and JMJD7 contain basic elements required for hydrolysis (Fig. 3C), such as similar residues chelating divalent ions (His321, Asp323, and His400 in JMJD5) and rich negatively charged sidechains as proton acceptor (Asp323 and imidic-Gln275 in JMJD5) within the catalytic center of JMJD5 (Fig. 2B, Fig. 3C)^34^. We found that methylated arginines play critical roles in the recognition between JMJD5/7 and methylated histone tails^8^. To investigate the basis of specific recognition and catalysis, we determined the complex structure of c-JMJD5 in the presence of a symmetric dimethyl arginine residue derivative (DM(s)-Arg), Zn^2+^, and α-KG (Fig. 3A,B,C, Table S4). Zn^2+^ substitutes functionally for Fe^2+^ (see below) and is more stable than Fe^2+^ in solution. We found that the methyl groups of di-methyl arginine are buried in a negatively charged pocket consisting of one glutamic acid (Glu238), two tyrosines (Tyr243, Tyr272), two tryptophans (Trp248, Trp310), one Ser318, and a portion of α-KG (Fig. 3C,D). This configuration is similar to the structure of Tudor domains bound by methylarginine residues (Fig. 3E and Fig. S5)^35–39^. The carboxyl group is coordinated with Zn^2+^; this structure could mimic the final step of peptidase cleavage with the oxygen of the carbonyl interacting with Zn^2+^ (Fig. 3D). To cross-confirm the authenticity, we also determined the complex structure of c-JMJD5 and monomethyl arginine, and to no surprise, the monomethyl arginine occupies the exact same position as that of dimethyl arginine, except missing a methyl group (Fig. S6, Table S5, Table S6). It will be of interest to obtain a complex structure of c-JMJD5 and asymmetric dimethyl arginine, given that there is enough space to hold two methyl groups at either side based on the large pocket within c-JMJD5. However, we failed to obtain crystals of c-JMJD5 with asymmetric dimethyl arginine or c-JMJD5 with any short peptide containing methylated arginines thus far.

### The catalytic mechanism of c-JMJD5 and c-JMJD7

All catalytic cores of JmjC proteins, or Fe^2+^ and α-KG dependent oxygenases, contain the highly conserved triple residues HXD/E(X)_n_H (X = any residue) to chelate Fe^2+^ (Fig. S3). This structural motif is highly similar to those of metallo-endopeptidases and metallo-exopeptidases^34^. Adding EDTA or mutating the three Fe^2+^-chelating residues to Ala completely abolished the enzymatic activities of c-JMJD5^8^. Interestingly, among metalloproteases, additional Glu, Asp, and His residues are required nearby for proton transfer, polarization, and nucleophilic attack of substrates. We hypothesized that the binding of α-KG by JmjC proteins may release some sidechains from three chelating residues and act as a part of a process similar to that enabled by electron rich residues in peptidases. In this regard, we tested the catalytic activities of c-JMJD5 and c-JMJD7 with and without α-KG. To avoid the contamination by α-KG, a point mutation of a critical α-KG binding lysine residue (Lys336) located at the bottom of the α-KG binding pocket (Fig. S5C, Fig. S6E) within c-JMJD5 to glutamic acid was generated. Interestingly, the mutation dramatically reduces the enzymatic activity of c-JMJD5 (Fig. 3F), suggesting that α-KG is essential for the catalytic activity of c-JMJD5.

For hydrolysis, only H_2_O (and not O_2_) is coordinated with divalent cations Fe^2+^ or Zn^2+^ in JmjC proteins and is polarized (Fig. 3C). We examined the sequences of JMJD5 and JMJD7 to investigate which residue is the intermediate proton acceptor during the catalysis process. We found that Gln275 in JMJD5 and Gln131 in JMJD7 are conserved and close to the catalytic center (Fig. 3C, Fig. S3). To test whether this residue is involved in the catalysis process, a point mutation of Gln275 to Ala was generated in c-JMJD5. The mutation nearly abolished the activity of c-JMJD5 (Fig. 3F). Although Gln275 is not a strong proton acceptor compared to Glu, Asp, or His, it may act as a proton acceptor during catalysis by c-JMJD5. Interestingly, several reports showed that amide-carbonyl groups of Gln and Asn can form an imidic acid to participate proton transferring under a special microenvironment^40–42^. A question here is why JMJD5 possesses glutamine instead of glutamic acid at position 275. To investigate the functional contribution of Gln275, a point mutation of Gln275 to Glu275 was generated. To our surprise, the Glu275 mutation has a higher enzymatic activity (Fig. 3G). We speculate that lower Gln275 activity may be required for JMJD5 *in vivo*. However, additional evidence is needed to prove this speculation.

### Specific binding between c-JMJD5/c-JMJD7 and substrates

**Figure 4:**
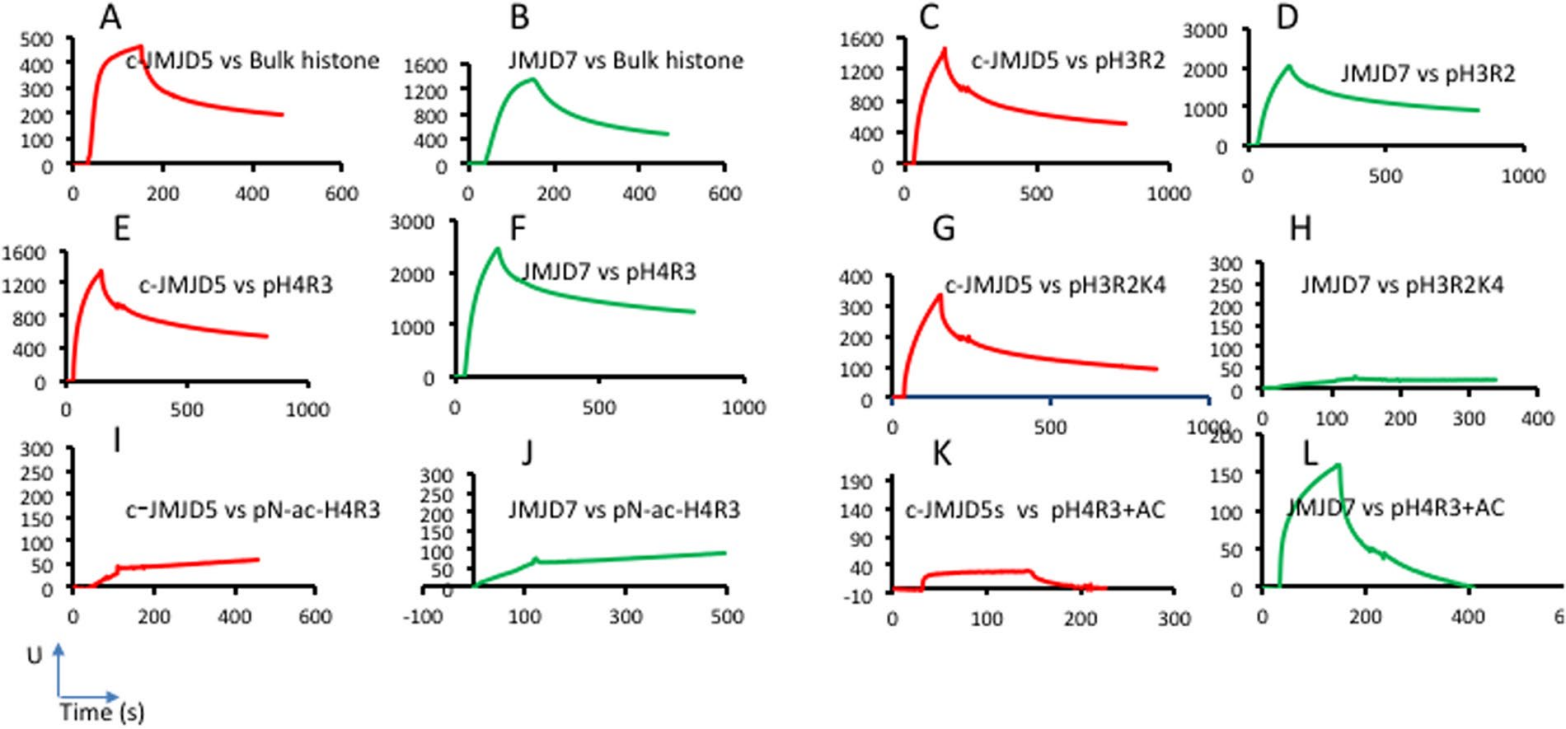
The specific binding between c-JMJD5/JMJD7 and bulk histone/synthetic peptides. A–L, are generated by Surface Plasmon Resonance Biacore, Y-axis, response RU, X-axis, time (seconds). M is generated by Fluorescence polarization, Y-axis, percentage of fluorescence intensity, X-axis, concentrations of peptides (μM). (**A**) c-JMJD5 specifically binds to bulk histone with significant binding affinity. (**B**) JMJD7 specifically binds to bulk histone with significant binding affinity. (**C**) c-JMJD5 binds to pH3R2me2a. (**D**) JMJD7 binds to pH3R2me2a. (**E**) c-JMJD5 binds to pH4R3me2a. (**F**) JMJD7 binds to pH3R4me2a. (**G**) c-JMJD5 binds to pH3R2(me2a)K4(me3). (**H**) JMJD7 fails to bind to pH3R2(me2a)K(me3). (**I**) c-JMJD5 fails to bind to pN-ac-H4R3(me2a)(N-terminal acetylated H4R3 peptide). (**J**) JMJD7 failed to bind to pN-ac-H4R3(me2a). (**K**) c-JMJD5 does not bind to pH4R3(me2a)K5(ac)K8(ac)K12(ac)K16(ac). (**L**) JMJD7 binds to pH4R3(me2a)K5(ac)K8(ac)K12(ac)K16(ac). Note: pH3R2 = pH3R2me2a, pH4R3 = pH4R3me2a, pH3R2K4 = pH3R2(me2a)K4(me3), pN-ac-H4R3 = pN-ac-H4R3(me2a), pH4R3 + AC = pH4R3(me2a)K5(ac)K8(ac)K12(ac)K16(ac), pH3R2 + AC = pH3R2(me2a)K4(ac)K9(ac)K14(ac)K18(ac).

The multiple reported activities of JMJD6 (e.g., lysine hydroxylase, arginine demethylase, and RNA demethylase) have raised questions concerning how this protein and related JmjC family members recognize cognate substrates^43^. Mechanisms that control substrate specificity are crucial towards understanding the functions of these proteins. Each of these proteins include residues necessary for similar biochemical reactions (*i.e*., oxidation of carbon-nitrogen bonds). Here, we assess a common criterion of specific and non-specific substrate recognition: the binding affinity between enzyme and substrate. In our previous studies of JMJD2 family members and their cognate peptide substrates, we identified binding affinities in the range of ~1 to 10 μM^29^. In preliminary experiments, we utilized bulk histones as substrates. Bulk histones are heterogeneous and theoretically contain most potential combinations of post-translational modifications. Thus, these substrates enable the detection of binding by different cognate enzymes. We immobilized bulk histones on a Biacore chip and injected the JmjC proteins to measure their binding affinity. We observed significant binding affinity of both c-JMJD5 and JMJD7 on bulk histones (due to non-linear binding kinetics, ranges of approximately 1–50 μM were estimated) (Fig. 4A,B). These binding affinity ranges are similar to those previously calculated between JMJD2 and its substrates. Although our data were obtained using complex ligands (i.e., bulk histones), these results suggest that c-JMJD5 and JMJD7 bind histones specifically.

To further confirm these conclusions, individual specific peptides of H3 (pH3) and H4 (pH4) with methylated arginines were synthesized and immobilized on Biacore chips for affinity measurements using c-JMJD5 or JMJD7 proteins. Both c-JMJD5 and JMJD7 bound to the pH3R2(me2) and pH4R3(me2) peptides with similar binding affinities (Fig. 4C–F, Table S7). These results are consistent with our previous data showing that both c-JMJD5 and JMJD7 digest methylated H3R2(me2) and H4R3(me2)^8^.

Cross-talk by histone binding proteins and modifiers of post-translational modifications controlled by the combination of histone modifications is a major epigenetic regulation mechanism^44^. For example, methylation of H3 tails at R2 and K4 (pH3R2K4) is recognized by the Recombination Activating Gene 2 (RAG2) protein^45,46^. Interestingly, we found that c-JMJD5 recognizes pH3R2K4 (Fig. 4G); however JMJD7 does not (Fig. 4H). This observation suggests differential preferences towards substrates by JMJD5 versus JMJD7, consistent with the differing charge distributions along the potential substrate binding areas between these two enzymes.

Acetylation on histone H4 is another important epigenetic modification. Although we detected binding of H4R3 peptide by c-JMJD5 or JMJD7 (Fig. 4E,F), neither c-JMJD5 nor JMJD7 bound to N-terminal acetylated H4R3 peptide (p-ac-H4R3) (Fig. 4I,J). Interestingly, c-JMJD5 completely loses binding affinity with respect to multiple site acetylation of H4R3 peptide at position K5, K8, K12, and pK16 (pH4R3(me2a)K5(ac)K8(ac)K12(ac)K16(ac)), while JMJD7 binds this peptide regardless of its additional acetylation state of pH4R3 (Fig. 4K,L). These properties further differentiate the two proteins from each other. To address the specificity of the native forms of histone peptides toward the two enzymes, native forms of H3 peptide (1–21, pH3) and H4 peptide (1–21, pH4) (Table S7) were subjected to binding assays. Unfortunately, due to non-specific binding, neither c-JMJD5 nor JMJD7 dissociated from the pH3 or pH4 attached channels after injection. All Surface Plasmon Resonance binding assays above not only reveal the specific binding of JMJD5/JMJD7 toward bulk histone and arginine methylated histone peptides, but also demonstrate the difference in substrate recognitions between JMJD5 and JMJD7. However, due to the non-linear binding property between enzymes and substrates, it is impossible to derive the exact binding constants using Surface Plasmon Resonance experiments. The following Fluorescence polarization experiments were introduced to resolve the exact binding constants between enzyme and substrates.

### Differential recognition of substrates with and without arginine methylation

The binding affinities of various lysine binding domains, such as Chromodomains, PHD fingers, Tudor domains, etc., to lysine residues with and without methylation has been very well established^47^. It is also true that the difference in binding affinity of Tudor domains to arginine with and without methylation is also marginal^39^. The question here is whether c-JMJD5 or JMJD7 has different binding affinity depending on the methylation status of arginine. From the complex structure shown above, c-JMJD5 contains a binding pocket for methylarginine that is similar to those of Tudor domains (Fig. 3D,E, Figs S5, S6). We reasoned that the rich aromatic environment in the binding pocket of c-JMJD5 could be sensitive enough for accurate binding affinity readout for both arginine containing peptides with and without methylation through fluorescence polarization experiments. This turned out to be true in our previous report, where we found that c-JMJD5 binds to native form pH3 peptide with a measurable binding affinity ~7 μM, to pH3R2me2a with surprisingly high affinity ~0.112 μM, or ~70 times that of pH3^8^. This binding affinity is similar to those between PHD domains and methylated lysines (0.16–30 μM), but is more than one order of magnitude higher than affinities between Tudor domains and methylated arginines (~4 μM)^39^. Based on the complex structure, much higher binding affinity between c-JMJD5 and pH3R2me2a (~0.1 μM) versus Tudor domains and methylarginine containing peptides (~4 μM) can be explained by the presence of additional charge-charge interactions within the methylarginine binding pocket of c-JMJD5 contributed by α-KG and a sidechain of glutamic acid (Glu238) (Fig. 3D). Interestingly, as pointed out by one of our reviewers, the sidechain of Ser318 makes a hydrogen bond with NE atom of sidechain of methylarginine (Fig. 3D), the binding should also contribute to the high binding affinity. Most importantly, this unique hydrogen bond may also differentiate methylarginine from methyllysine. Further investigation is needed.

Our previously reported results showed that c-JMJD5 discriminates pH3R2me2a from pH3 by exhibiting a much higher binding affinity. Although the biochemical mechanism in which c-JMJD5 discriminates between methylated versus unmodified arginine is unknown, it was demonstrated using lysine with various methylation states to assert that the methyl-π hydrophobic interactions within the aromatic cage is responsible for the observed higher affinity among higher lysine methylation states compared to unmodified lysine or lower lysine methylation states^48^. Pending further investigation, this explanation fits our observation that pH3R2me2a exhibits greater binding affinity, compared to pH3, within c-JMJD5’s aromatic cage as well. Taken together, the dramatic differences in binding affinity between c-JMJD5 and histone tails with or without arginine methylation further confirms that JMJD5 is more specific for sites with arginine methylation.

**Figure 5:**
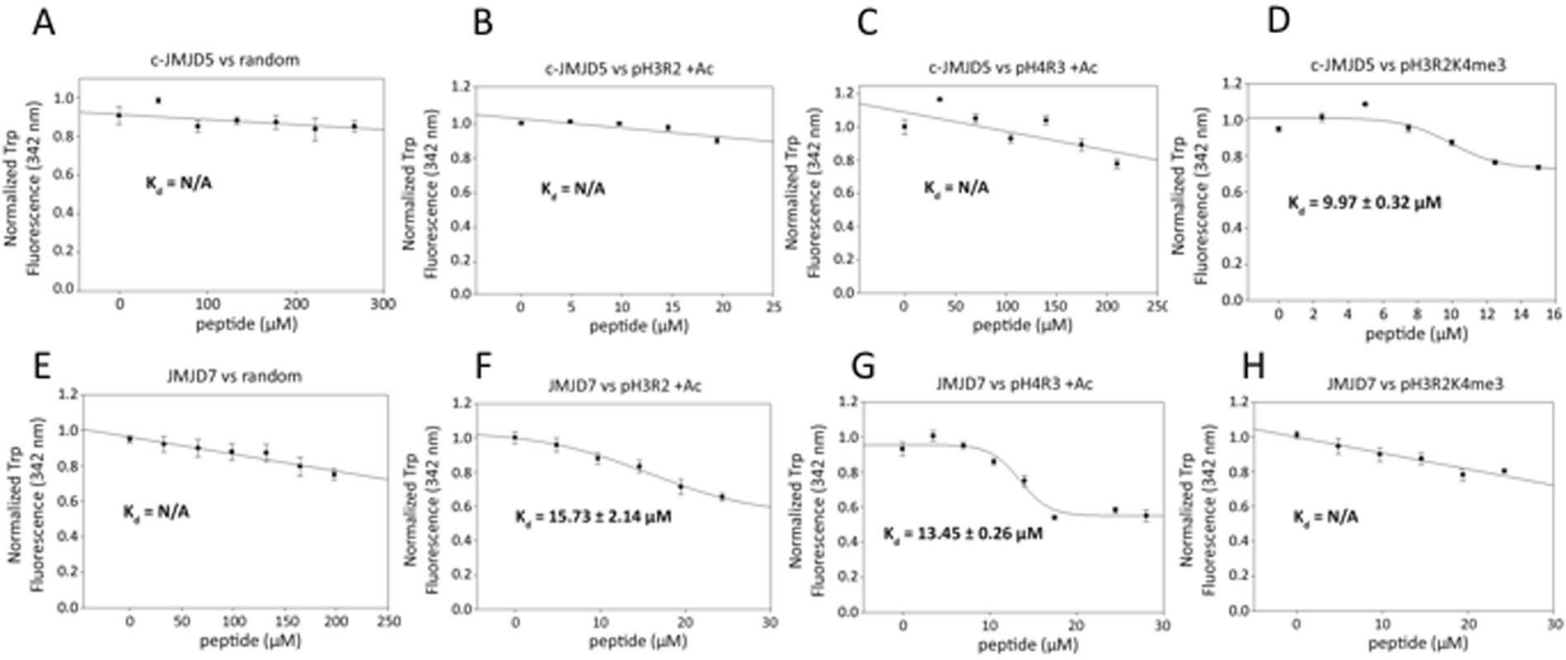
Fluorescence polarization anisotropy binding assays of c-JMJD5 and JMJD7 to different peptides. (**A**) The mixture of c-JMJD5 to a random peptide shows no binding. (**B**) The mixture of c-JMJD5 to pH3R2(me2a) with additional acetylation of all available lysine residues results in no binding (pH3R2 + AC). (**C**) The mixture of c-JMJD5 to pH4R3(me2a) with additional acetylation of all available lysine residues results in no binding (pH4R3 + AC). (**D**) The mixture of c-JMJD5 to pH3R2(me2a)K4(me3) peptide results in binding constant 9.97 ± 0.32 μM. (**E**) The mixture of JMJD7 to a random peptide shows no binding. (**F**) The mixture of JMJD7 to pH3R2(me2a) with additional acetylation of all available lysine residues results in binding constant 15.73 ± 2.14 μM (pH3R2 + AC). (**G**) The mixture of JMJD7 to pH4R3(me2a) with additional acetylation of all available lysine residues results in binding constant 13.45 ± 0.26 μM (pH4R3 + AC). (**H**) The mixture of JMJD7 to pH3R2(me2a)K4(me2) peptide results in no binding.

From Surface Plasmon Resonance binding assays, c-JMJD5 and JMJD7 seem to recognize different combinations of modification of histone tails (Fig. 4). To further confirm the results, two forms of peptides with additional methylation and acetylation respectively on the basis of original pH3R2me2a and pH4R3me2a were subjected to fluorescence polarization binding assays. As expected, c-JMJD5 does not bind to either pH3R2me2a or pH4R3me2a peptides with additional acetylation (pH3R2me2a +AC, pH4R3me2a +AC) (Fig. 5B,C), while JMJD7 binds them with affinities of 15.73uM and 13.45uM, respectively (Fig. 5F,G). On the other hand, c-JMJD5 binds to pH3R2K4me3 with a binding affinity of 9.97uM (Fig. 5D) while JMJD7 does not (Fig. 5H). These data suggest that JMJD5 and JMJD7 differentiate themselves from recognizing different combinations of modification of histone tails. This is also reflected by the different charge distribution around catalytic cores of both enzymes as showed in the structural section. However, we must be aware that all these data are generated from short peptides, they may not reflect actual situation of histone tails within nucleosomes *in vivo*.

## Discussion

Clipping of histone tails, high turnover rates of histone in non-proliferating cells, arginine demethylation, and Pol II elongation control are highly contentious subjects in the transcription and epigenetic fields. Our previous novel discovery of clipping of arginine methylated histone tails by JMJD5 and JMJD7 sheds light on how these topics are intrinsically coupled^8^. Data presented here not only reveal the underlying mechanism of novel enzymatic activities of JMJD5 and JMJD7, but also further corroborate our previous findings. Shortly after the release of our report, Shen *et al*. also reported the proteolytic activity of JMJD5^49^. That report represents a strong vindication of our observations, given that at least two separate research groups independently discerned JMJD5’s role as a protease that specifically cleaves histone tails. Although the Shen *et al*. report is only limited to methyl-lysines on H3 tail as cleavage targets, this may reflect JMJD5’s potentially broad substrate specificity beyond just methylarginine. In our previous report, JMJD5 has slightly increased binding affinity toward H3K4 methylated peptide (pH3K4(me2), ~4 μM) versus native form H3 (pH3, ~7 μM)^8^. At the same time, Tudor domains can recognize both methylated arginines and lysines. Following this rationale, the Tudor-domain-like structure of JMJD5 should accommodate both methylated arginines and lysines as well. However, the hydrogen bond between NE atom of methylarginine and the sidechain of Ser318 could be critical for substrate discrimination. Nevertheless, further characterization is needed to determine whether JMJD5 and JMJD7 have similar binding affinities towards methylated arginines or lysines on other sites of histone tails.

**Figure 6:**
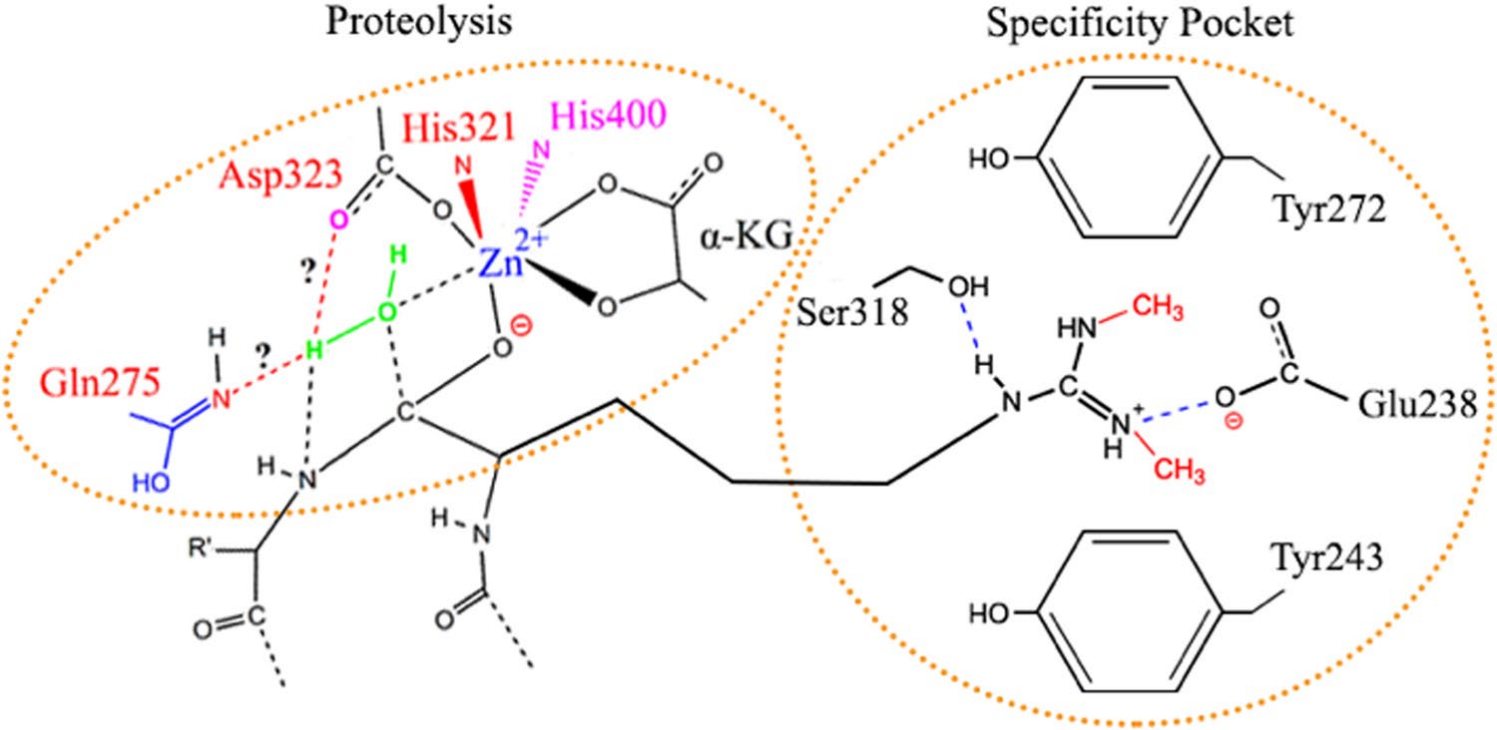
Proposed model of the proteolytic hydrolysis mechanism of JMJD5. The methylated sidechain of Arginine docks at the hydrophobic and negatively charged pocket, which brings the peptide bond of the target to close proximate of Zn^2+^ and α-KG. Polarization of water molecule and the peptide bond lead to the rearrangement of individual groups to cleave the peptide bond. Gln275 and Asp323 could act as intermediate proton “H” acceptor (potential through imidic acid intermediate). Specificity pocket comprised of Tyr272, Tyr 243, Glu238, and Ser318 can accommodate side chains of Arginine residue with or without methylation, including mono-, di-asymmetric-, di-symmetric-.

An interesting evolutionary question concerns how a family of oxygenases can give rise to proteases with both metallo-endopeptidase and metallo-exopeptidase activities. According to established catalysis mechanisms of these enzyme families, Zn^2+^, which is coordinated by conserved histidine residues, together with aspartic acid or glutamic acid, are key elements that polarize the carbonyl groups of substrate peptides and water molecules (Fig. 3C, Fig. 6). All JmjC proteins including JMJD5 and JMJD7 contain this unique structural motif, which is coordinated by Zn^2+^ (Fig. 3C). From our activity assays, adding EDTA or mutating the three ligation residues abolished the activities of c-JMJD5 and JMJD7, suggesting the critical roles of divalent cations and coordinating residues^8^. Interestingly, our proteolytic assays showed that switching Fe^2+^ to Zn^2+^ does not change the protease activity of either c-JMJD5 or JMJD7, suggesting that the catalytic activities of the enzymes does not require electron exchange as observed in hydroxylation. We expect that the abundance of Zn^2+^ ions in the nucleus is conducive for the activities of JMJD5 and JMJD7. However, because JmjC proteins possess conserved structures and cofactors, it remains intriguing as to how JMJD5 and JMJD7 evolved to perform proteolytic activities in higher eukaryotes. One possible explanation is that a functional switching mechanism is employed under hypoxic environments.

In addition to divalent cations chelating residues, all metallo-endopeptidases and metallo-aminopeptidases require additional residues for their complete enzymatic activities. From the structures of c-JMJD5 and JMJD7, we found that one glutamine residue in each enzyme (Gln131 in JMJD7 and Gln275 in JMJD5) is close to the catalytic center and likely plays a critical role as a proton transferring intermediate (Fig. 3C–F). The mutation of Gln275 to Ala nearly abolished the activity of c-JMJD5 while the mutation of Gln275 to Glu increases the activity of c-JMJD5 (Fig. 3F,G). These data confirm the hypothesis that this residue is important for catalysis. However, we did not find similar structures in other JmjC proteins with defined hydroxylase activities. Therefore, we hypothesize that glutamines in JMJD5 and JMJD7 confer the proteolytic activities of JMJD5 and JMJD7. Although Gln275 is not a strong proton acceptor compared to Glu, Asp, or His, it was reported that the carboxamide of Asn could form imidic acid to participate in proton relay in hydrolases, as well as SIRT2 deacetylase family proteins^40–42,50^.

All JmjC proteins examined required α-KG as a co-factor for catalysis in the hydroxylation process. For hydroxylation, α-KG is converted to succinate with release of CO_2_. From the complex structure of c-JMJD5 and the methylated arginine derivative (Fig. 3), it is clear that α-KG is not only involved in pocket formation, but also forms a salt bridge or hydrogen bond with the methylated guanidine group (Fig. 3D). α-KG also participates in the chelation of Zn^2+^ ions (Fig. 3C). Loss of α-KG binding due to a point mutation of lysine to glutamic acid also causes a huge loss of the activity of c-JMJD5. However, the exact roles of α-KG in JMJD5 and JMJD7 are not clearly defined. α-KG may participate directly in substrate binding, direct catalysis, or both. On the other hand, the joining of α-KG to chelate divalent ions also reduce the close association of the sidechain Asp323 toward the divalent ion (Fig. 3C), which may confer the sidechain of Asp323 the role of proton acceptor during hydrolysis. Nevertheless, based on those information, we can propose a potential hydrolysis mechanism that includes the following steps (Fig. 6). First, the sidechain of methylated arginine docks at the negatively charged pocket with aromatic cage, bringing the carbonyl group of this residue within close proximity to Zn^2+^. Second, the coordination of basic elements of hydrolysis including, Zn^2+^, Gln275, and potentially Asp323 of the JmjC domain with the peptide bond of the target, polarizes H_2_O (Fig. 6). Third, rearrangement of these elements results in cleavage of the peptide bond (Fig. 3C and Fig. 6). Interestingly, replacement of Fe^2+^ with Zn^2+^ or Co^2+^ does not affect the activities of c-JMJD5 (Fig. S7).

 Analysis of products after reactions of c-JMJD5 and JMJD7 with synthesized peptides, we found that c-JMJD5 and JMJD7 preferentially cleaved methylarginine residues, but also continued cleavage after the initially targeted residue and lysine residues^8^. Thus, after the first excision, the enzyme continued to act as a progressive aminopeptidase, resulting in digestion of the remaining C-terminal peptide. The region around the catalytic cores of JMJD5 and JMJD7 contain an area with a relatively hydrophobic and negatively charged space that could accommodate methylated and positively charged residues (Fig. 3D). The binding properties of JMJD5 and JMJD7 are similar to those of PHD and Tudor domains, or other methylated lysine and arginine binding domains. After the first cleavage and with the N-terminal fragment juxtaposed with the catalytic core, any positively charged, neutral, or hydrophobic residue at the N-terminal of a C-terminal product will be a substrate after a minor positional movement and will be exposed to further cleavage.

*In vitro* experiments are carried out under high concentrations of both enzymes and substrates, which may lead to artificial or non-specific results. In this regard, we must examine binding affinity to judge whether the enzyme and substrate is cognate or specific. Both c-JMJD5 and JMJD7 bind to bulk histone with moderate binding affinity ranges around ~1–10uM (Fig. 4A,B) suggesting specific recognition between JMJD5/7 and bulk histone tails. Since a combination of histone tail modifications is a common phenomenon, we reasoned that other modifications such as acetylation and methylation of neighboring residues required for the binding of both enzymes. Interestingly, neither c-JMJD5 nor JMJD7 binds to H4R3 with acetylation at the N-terminus. More interestingly, JMJD7 binds to hyperacetylated pH4R3 and pH3R2, but c-JMJD5 does not. Additional methylation of H3R2 N-terminal tails of H3 is also a common phenomenon. Methylation on K4 (pH3R3K4me3) prevents the binding of JMJD7, but not c-JMJD5 (Figs 4, 5). These data suggest that c-JMJD5 and JMJD7 share common substrates but also recognize some specific substrates individually. Further characterization of specificity of substrates for both c-JMJD5 and JMJD7 is required to demonstrate the preference of each enzyme. The most satisfying data are from our experiments utilizing fluorescence polarization, which showed that c-JMJD5 prefers pH3R2 over pH3 or pH3K4^8^. This data further support our hypothesis that JMJD5 specifically recognizes peptides with methylated arginines. Additionally, JMJD5 does not bind to acetylated histone tails, which is a hallmark for nucleosomes within active genes. An extended speculation is that JMJD5 may only work on nucleosomes at position +1, which are very well characterized as an obstacle for Pol II elongation among stimulating genes^7,11^. This result further support our previous hypothesis that JMJD5 cleave histone tails on nucleosomes at position +1 to generate “tailless nucleosomes” for Pol II to overcome without additional assistance^8^.

In brief, we have determined structures of c-JMJD5 and JMJD7 under different conditions. In particular, the structure of c-JMJD5 was determined with and without substrates. c-JMJD5 is highly similar in 3D structure but have dramatic differences in charge distribution near their catalytic centers compared to JMJD7. These differences in charge are responsible for determining substrate specificity. c-JMJD5 and JMJD7 each contain critical structural elements responsible for both endopeptidase and exopeptidase activities. Furthermore, both JMJD5 and JMJD7 have specific binding affinity toward arginine methylated histone tails. Most importantly, c-JMJD5 prefers additional methylated histone tails while JMJD7 prefers additional acetylated histone tails.

## Materials and Methods

### Crystallization, Data Collection, structural determination, and refinement of JMJD5, MBP-JMJD7 and JMJD7

Both human version of c-JMJD5 and mouse version of JMJD7 are expressed and purified as previously reported^8^. c-JMJD5 with and without dimethylarginine/monomethylarginine was crystallized by vapor diffusion in hanging drops with 0.1 M HEPES pH7.0, 8% PEG3350 at 8 ^°^C with 1 mM α-KG and 1 mM Zn^2+^added. For data collection, c-JMJD5 crystals were transferred to a cryo-protecting buffer (reservoir buffer supplemented with 20% glycerol) and frozen in liquid nitrogen. All data of c-JMJD5 with and without substrates used in structure solving and refinement were collected on a beam line 24-ID-E, APS, Argonne National Laboratory. Data were integrated and scaled using the HKL2000 suite of programs^51^. Structural determination and refinement results are shown in Tables S1, S5, S6.

MBP-JMJD7 (20 mg/ml) (mouse JMJD7) was crystallized by vapor diffusion against 100 mM Citrate Sodium, 20% PEG 3350 (Hampton Research) at 4 °C. For data collection, MBP-JMJD7 crystals were flash frozen in liquid nitrogen and sent to a synchrotron. The native JMJD7 crystals diffracted to only 9~10 Å using a home light resource, so further dehydration was performed by increasing the PEG concentration in the well reservoir. After dehydration, the diffraction of JMJD7 was increased to 4.1 Å with the home light resource. JMJD7 crystals were frozen in liquid nitrogen and sent to a synchrotron. All data used in structure solving and refinement were collected on a beam line 4.2.2 (MBC-ALS) at the Advanced Light Source (Berkeley, ALS, USA). Data collection statistics are shown in Table S2. Data were integrated and scaled with the XDS Program Package^52^. Structural determination and refinement results are shown in Table S2.

mJMJD7 (generated from GST-mJMJD7 and affinity-tag removal, 10 mg/ml) was crystallized by vapor diffusion against 0.1 M MES monohydrate pH6.0, 10% PEG4000 at 4 °C. For data collection, mJMJD7 crystals were flash frozen in liquid nitrogen and sent to a synchrotron. All data used in structure solving and refinement were collected on beam line 4.2.2 (MBC-ALS) at the Advanced Light Source (Berkeley, ALS, USA). Data collection statistics are shown in Table S3.

### Biacore binding of JMJD5 and JMJD7 to bulk histone or synthetic peptides

10 μg/ml calf bulk histone was dissolved in coating buffer (50 mM sodium acetate [pH 5.0]) and coated onto a CM5 chip (GE Healthcare). Different concentrations of JMJD7 protein were then injected to calculate the binding affinity between bulk histone and JMJD7. Peptides were ordered from AnaSpec Inc. The sequence for the peptides used in our experiment are listed in Table S7. For peptides, 2 μg/ml synthetic peptide with C-terminal biotin was dissolved in PBS and coated onto a streptavidin coated SA chip (GE Healthcare). Different concentrations of JMJD7 protein were then injected to calculate the binding affinity between peptide and JMJD7. Similar procedures were carried out for JMJD5. All experiments are carried out at room temperature.

### Fluorescence Polarization experiment

Given the known structure of JMJD5, where aromatic residues are present in close proximity to the binding pocket, the following tryptophan fluorescence assay was deemed suitable for characterizing various peptide binding activity. All the regular and methylated peptides were synthesized by AnaSpec Inc. 10 μM c-JMJD5/JMJD7 was titrated and equilibrated with fixed concentrations of each peptide respectively, incubated at 25 °C for 30 min between each titration intervals, and subject to fluorescence measurement. The buffer used in the fluorescence quenching assay was 20 mM Tris-HCl pH 6.5. The excitation wavelength of 280 nm and the emission wavelength of 342 nm was used for data collection and recorded with a Fluoromax-3 spectrometer. The titration samples were prepared and analyzed in parallel between 2 to 4 times. All values at different titration points were compiled, normalized against the maximum value obtained prior to titration and averaged. The error bars indicate the normalized minimum and maximum values at any given titration point. The K_D_ for each peptide was calculated by fitting to a 4 parameter sigmoidal dose-response curve with SigmaPlot v11.0.

### Enzymatic assays with Radiolabeling of bulk histone with PRMTs using 14C-SAM

Overall, all activity assays of mutated versions of c-JMJD5 and JMJD7 follow the similar procedures as reported^8^. Briefly, bulk histone treated with PRMTs were used as substrates for mutated c-JMJD5 and JMJD7. After the reaction, the sample was separated by SDS-PAGE gel, which was then dried using a vacuum pump. The ^14^C radioactive signal was detected using a Typhoon 9500 imager (GE Healthcare).

## Electronic supplementary material

Supplementary figures and tables
