# Supplementary material for: Specific Recognition of Arginine Methylated Histone Tails by JMJD5 and JMJD7
Source: Sci Rep. 2018 Feb 19;8:3275. doi: 10.1038/s41598-018-21432-8 (PMC5818494; doi:10.1038/s41598-018-21432-8)
Supplement: Supplementary file 1 — Supplementary figures and tables [file 41598_2018_21432_MOESM1_ESM.pdf]

Specific Recognition of Arginine Methylated Histone Tails by JMJD5 and JMJD7

- Haolin Liu[1,5,6,*], Chao Wang[1,5,*], Schuyler Lee[1,5*], Fangkun Ning[1,5,], Yang Wang[1,5], Qianqian Zhang[2], Zhongzhou Chen[2], Jianye Zang[3], Jay Nix[4], Shaodong Dai[1,5], Philippa Marrack[1,5,6], James Hagman[1,5], John Kappler[1,5,6], Gongyi Zhang[1,5,#]
-
- [1]Department of Biomedical Research, National Jewish Health, 1400 Jackson St, Denver, CO 80206, USA; [2]State Key Laboratory of Agrobiotechnology, Chinese Agricultural University, Beijing 100193, P. R. China; [3]Department of Molecular Biology, University of Science and Technology of China, Hefei, 900015, P. R. China; [4] Molecular Biology Consortium, Advanced Light Source, Lawrence Berkeley National Laboratory, Berkeley, California 94720, USA; [5]Department of Immunology and Microbiology, School of Medicine, University of Colorado Denver, Denver, CO 80206, USA; [6]Howard Hughes Medical Institute, Denver, CO 80206, USA.
- *Contributed equally to the work
- #Corresponding authors: Gongyi Zhang: zhangg@NJhealth.org. Coordinates of JMJD5 and JMJD7 have been deposited at RCSB PDB Bank as: 4QU1, 4QSZ, 4QU2, 6AX3, 6AVS, 5FBJ

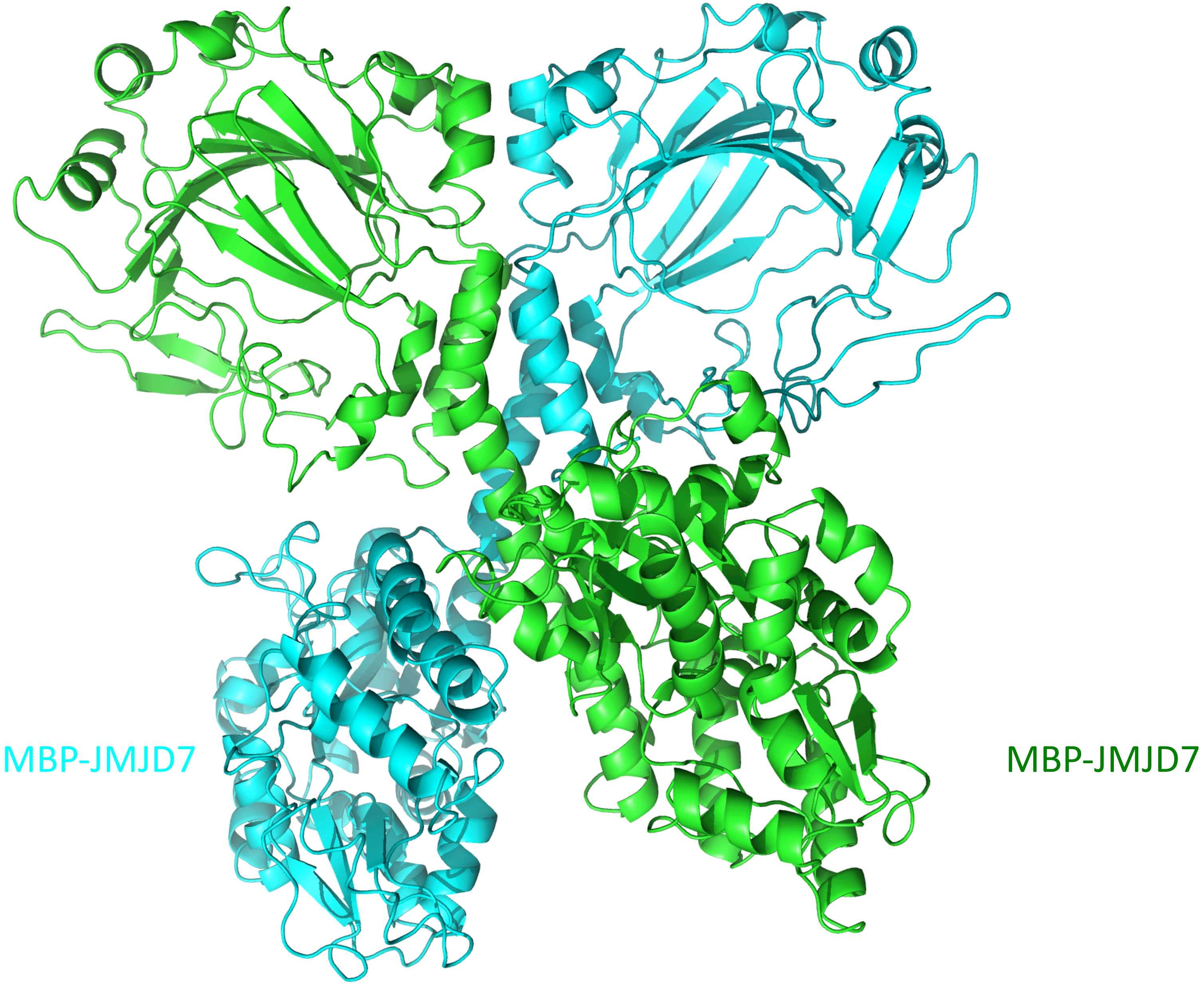

Figure S1. The dimer structure of MBP-JMJD7 in crystal

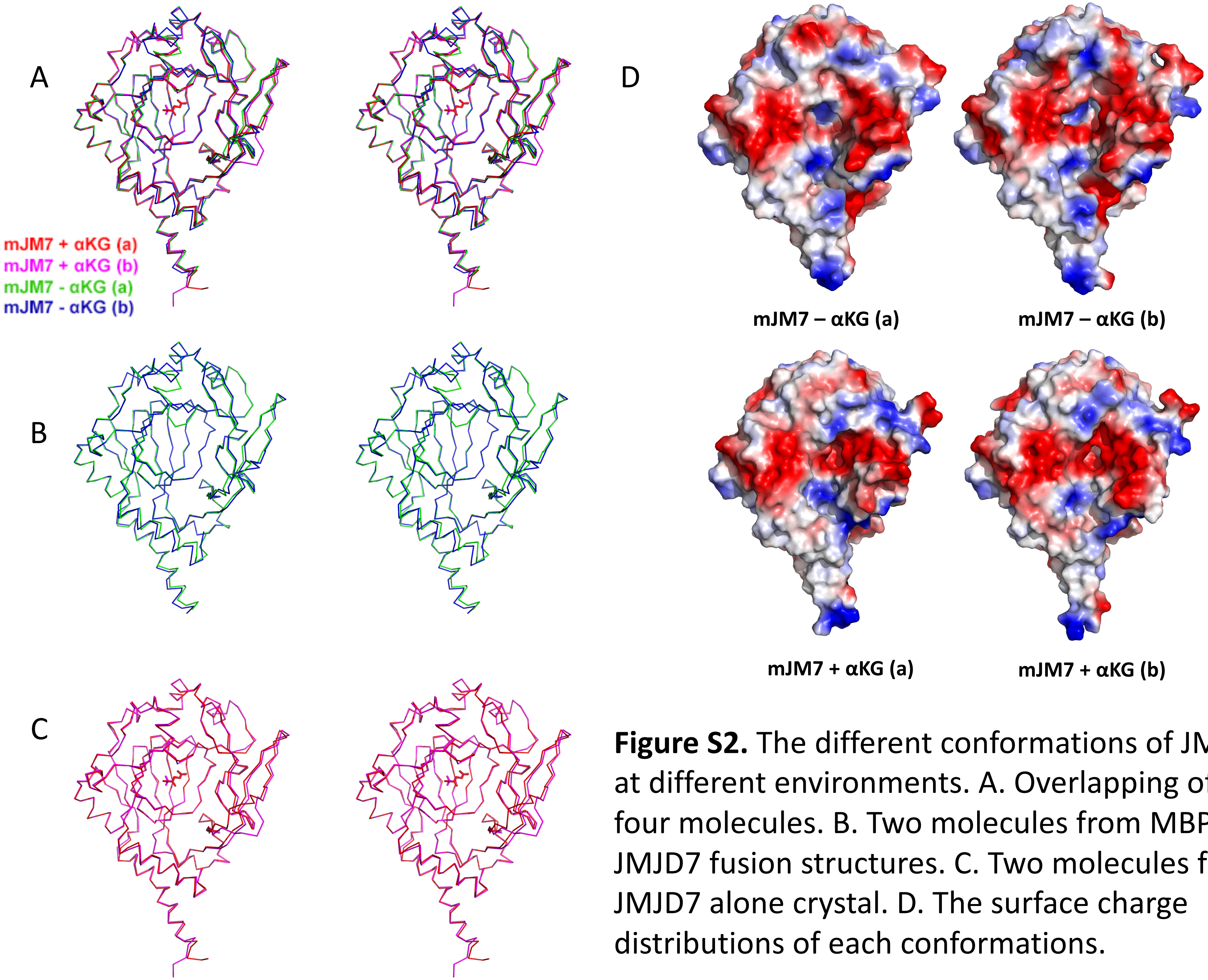

Figure S2. The different conformations of JMJD7 at different environments. A. Overlapping of all four molecules. B. Two molecules from MBP-JMJD7 fusion structures. C. Two molecules from JMJD7 alone crystal. D. The surface charge distributions of each conformations.

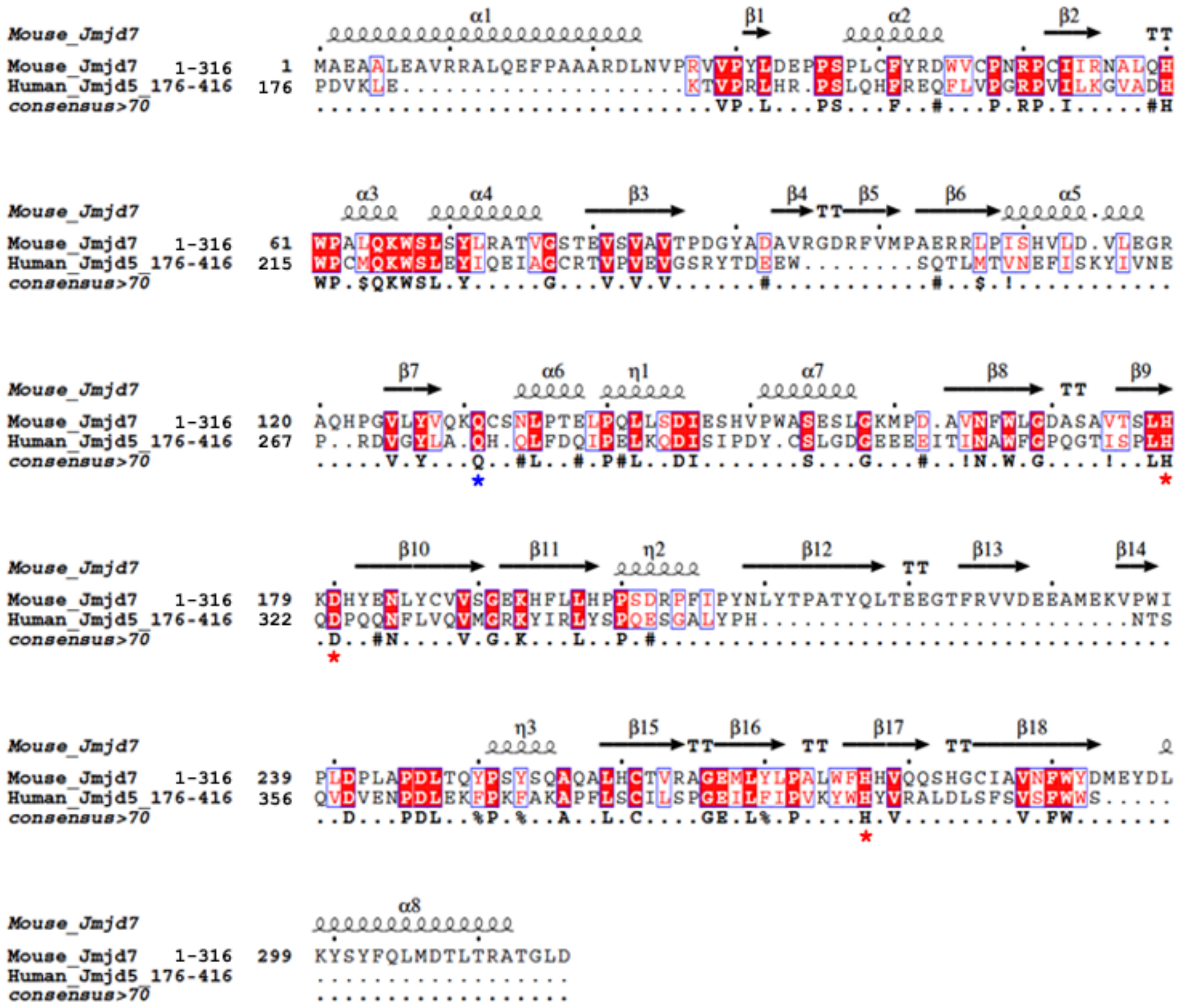

Figure S3. The sequence alignment of JMJD5 and JMJD7 on the basis of 3D structures. Q275 of JMJD5 and Q131 of JMJD7 are marked with blue star. HXD/E…H are marked with red stars.

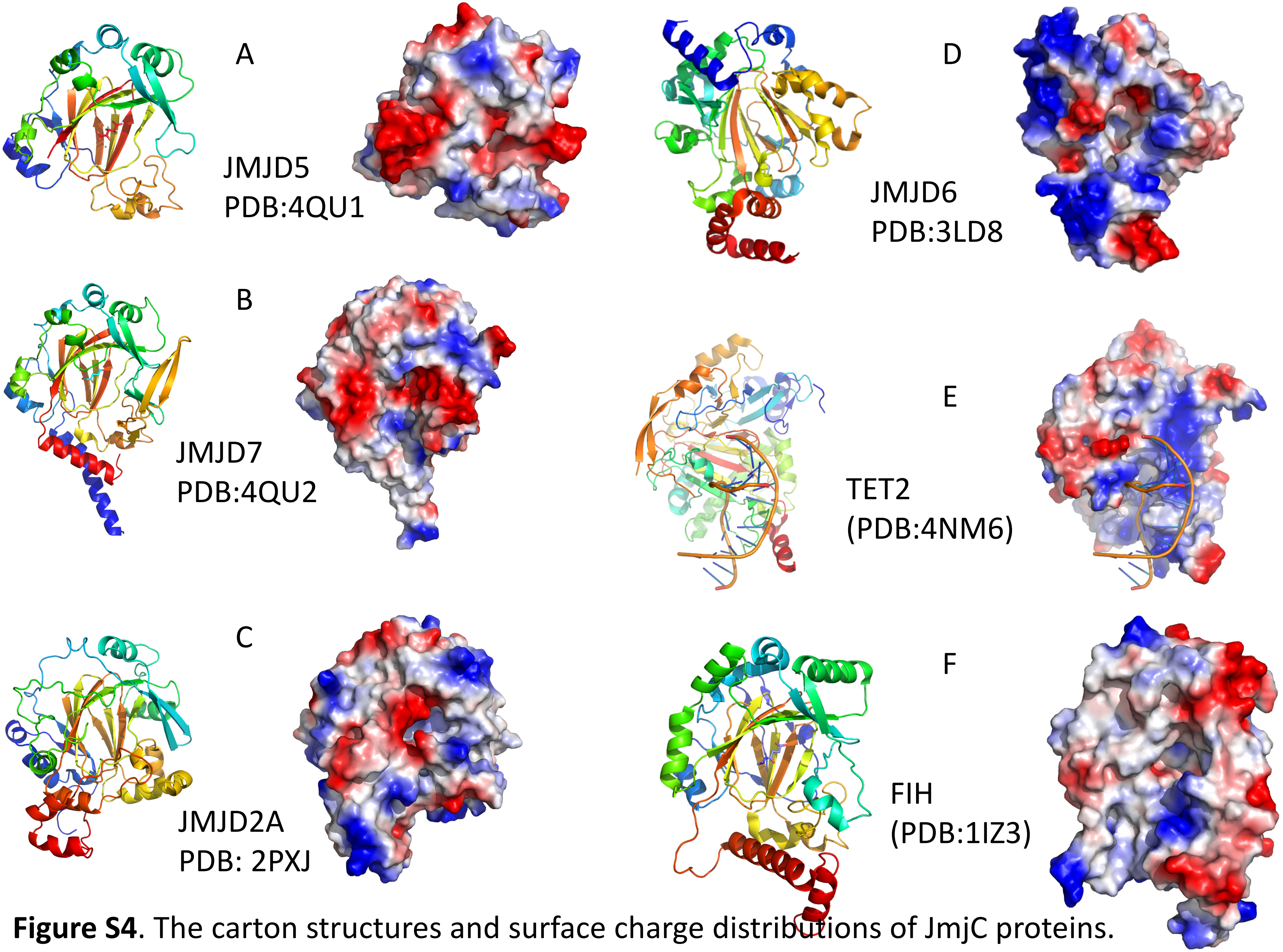

Figure S4. The carton structures and surface charge distributions of JmjC proteins. A. c-JMJD5. B. JMJD7. C. JMJD2A. D. JMJD6. E. TET2. F. FIH.

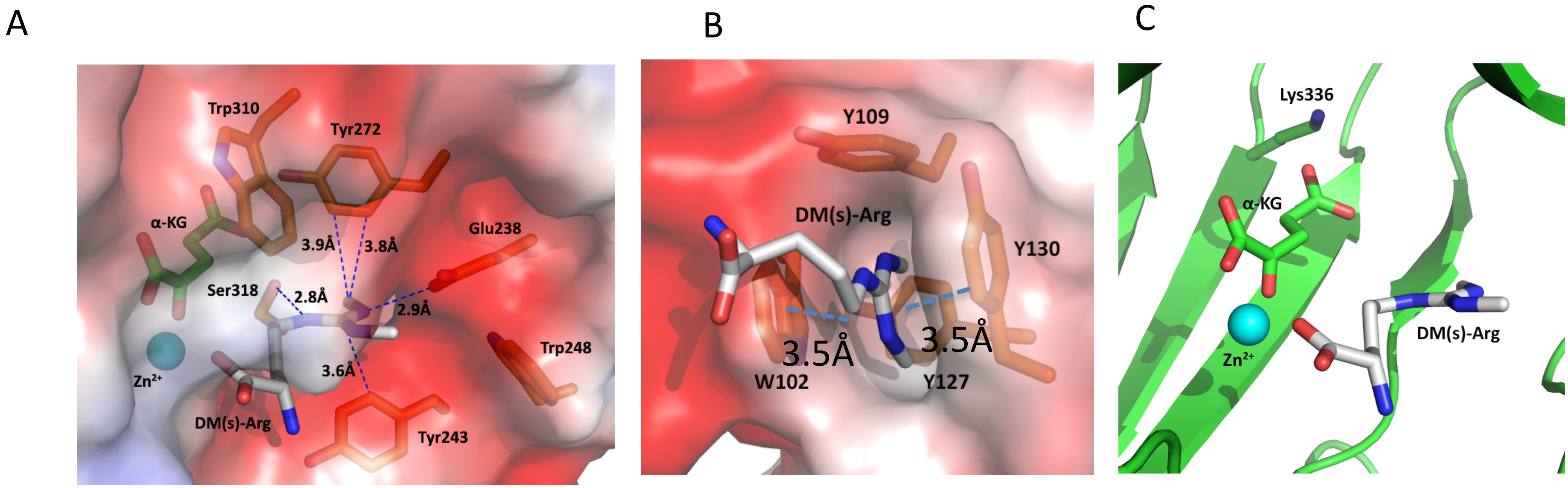

Figure S5. The similarity between c-JMJD5 substrate binding pocket and Tudor Domain from protein SMN (PDB ID: 4A4E). A. Complex of c-JMJD5 and a symmetric dimethyl-Arg. B. Tudor domain and a symmetric dimethyl-Arg. C. Lys336 is critical for binding of α-KG in c-JMJD5 with dimethylarginne (DM(s)-Arg).

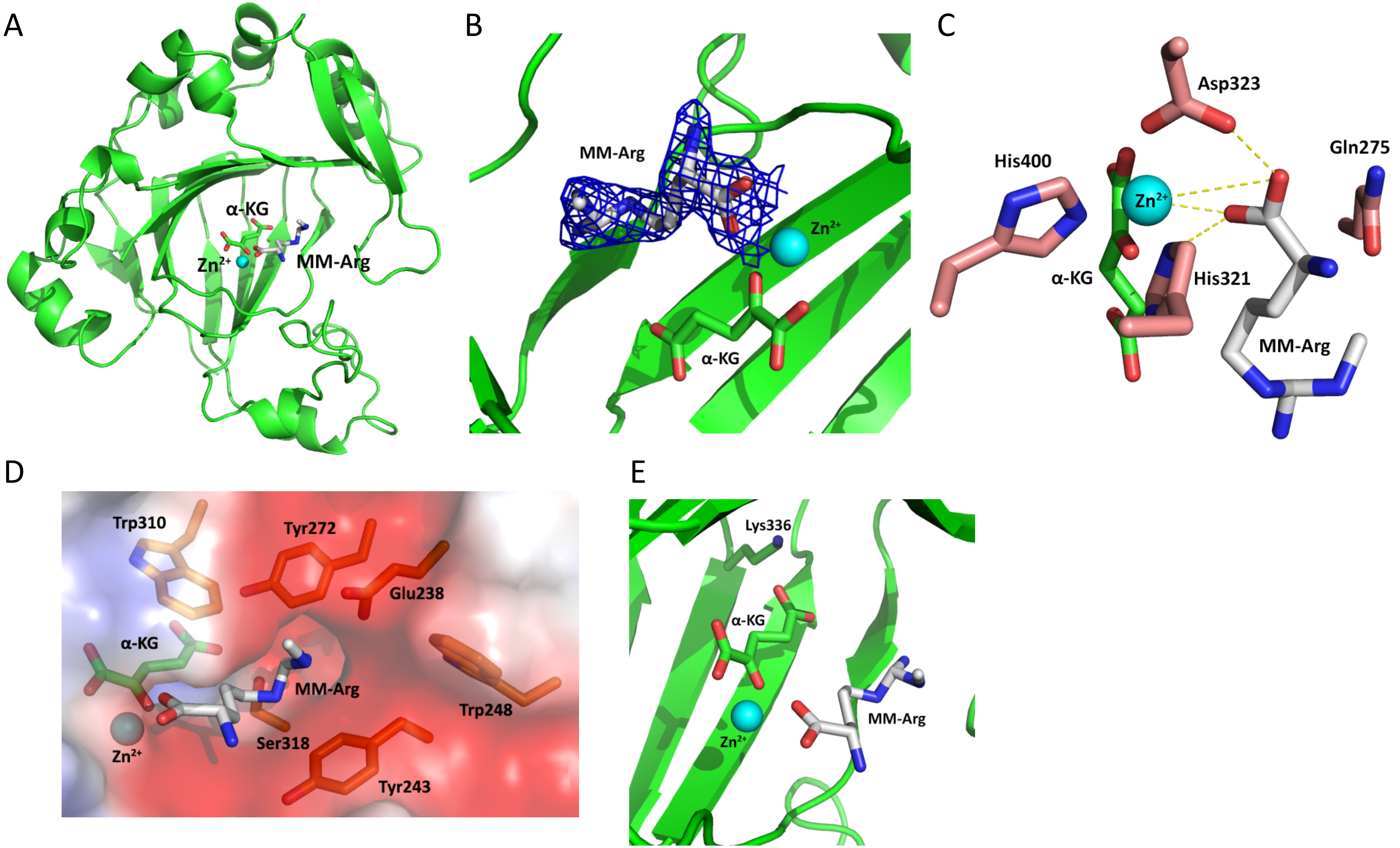

Figure S6. The complex structure of c-JMJD5 and a monomethyl arginine (MM-Arg). **A.** Complex structure of MM-Arg and JMJD5. **B.** 2Fo-Fc electron density of MM-Arg before refinement. **C.** The coordination of elements at catalytic center. **D.** The binding pocket for methylated guanidine group of arginine. **E.** Lys336 is critical for binding of α-KG in c-JMJD5. MM-Arg, monomethyl arginine.

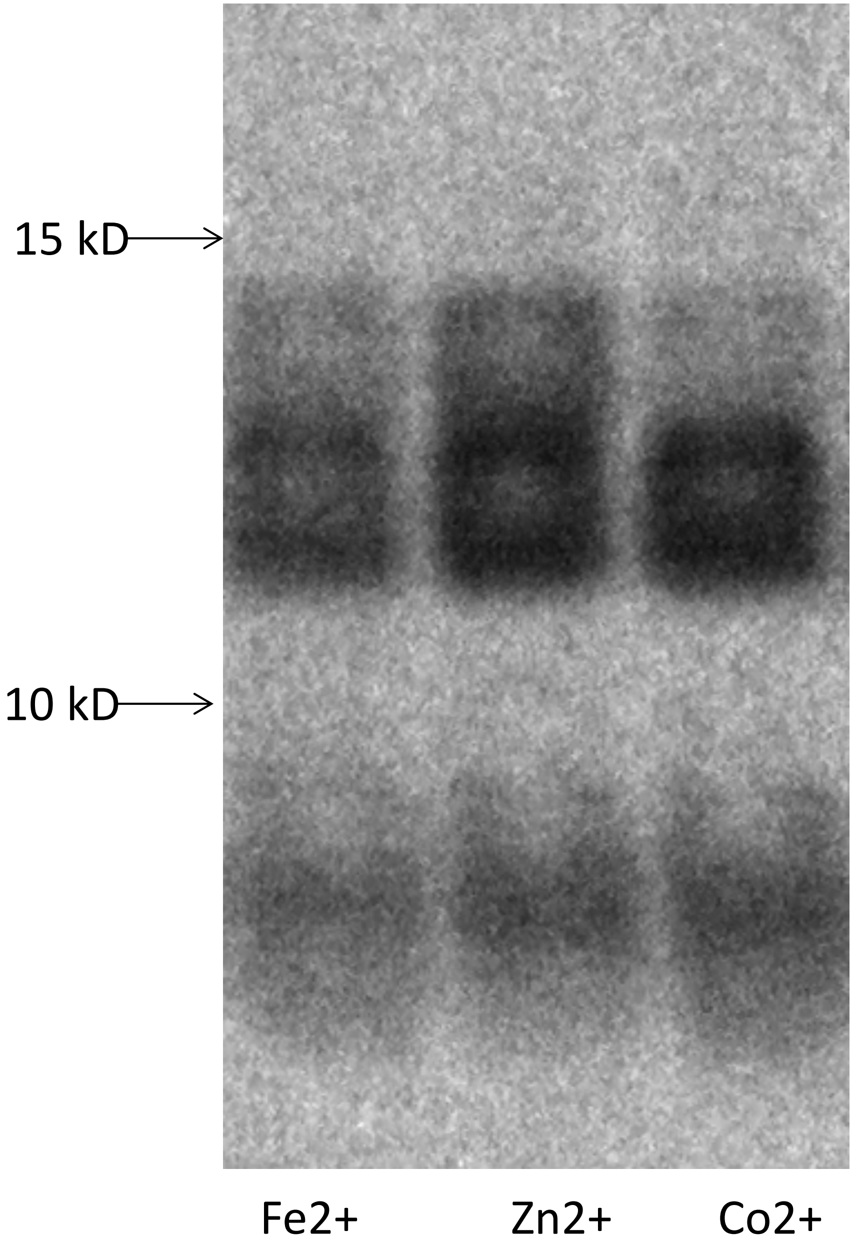

Figure S7. PRMT1 generated bulk histone sample is incubated with c-JMJD5 at presence of Fe^{2+}, Zn^{2+}, and Co^{2+} at pH7.0.

Table S1. Structure determination and refinement of hJMJD5 (PDB:4QU1).

There are many hJMJD5 structures available in the PDB bank, here we used 4GAZ as initial model to perform molecular replacement using PHASER and PHENIX refinements. COOT program was used for manual model adjustment. The final model of hJMJD5 contains 236 amino acid residues, 1 ION and 1 a-KG. The model was refined to 1.57 angstrom with R/Rfree of 14.9/17.4. Model quality was checked with the PROCHECK program (https://www.ebi.ac.uk/thornton-srv/software/PROCHECK/).

**Data collection**	
Wavelength (Å)	1.0000
Space Group	$P2_12_12_1$
Resolution (Å)	41.85-1.57 (1.65-1.57)[a]
Cell parameters	
a (Å)	49.627
b (Å)	65.030
c (Å)	77.782
Observed reflections	67,807
Unique reflections ($I/\sigma(I) > 0$)	35,833
Average redundancy	7.1 (6.1)
Average $I/\sigma(I)$	20.2 (2.4)
Completeness (%)	100.0 (99.9)
R_{merge} (%)[b]	8.28 (9.40)
**Refinement**	
Resolution (Å)	41.85-1.57
Working set/test set	35,767/3,577
R_{work}/R_{free} (%)[c]	14.90/17.40
No. of protein atoms	2,240
No. of water atoms	279
Root mean square deviation	
Bond lengths (Å)	0.006
Bond angles (degrees)	1.042
Ramachandran plot (%)	
Most favored regions	90.1
Allowed regions	9.4
Disallowed regions	0

[a] Numbers in parentheses refer to the highest resolution shell.
[b] $R_{merge} = \sum_{hkl}\sum_i |I_i(hkl) - \langle I(hkl)\rangle| / \sum_{hkl}\sum_i |I_i(hkl)|$ where $I_i(hkl)$ is the intensity of the ith measurement, and $\langle I(hkl)\rangle$ is the mean intensity for that reflection.

[c] $R_{factor} = \sum\sum_{hkl}||Fo|-|Fc||/\sum\sum_{hkl}|Fo|$, where |Fo| and |Fc| are the observed and calculated structure factor amplitudes, respectively.
.

Table S2. Structure determination and Refinement of MBP-mJMJD7 (PDB: 4QSZ).

MBP (PDB ID: 1HSJ) and c-JMJD5 model (PDB ID: 4QU1) were used as the initial model to perform molecular replacement with PHASER. There are two molecules in asymmetric unit. PHENIX Auto-Build was used to build model and improve the density map. COOT program was used for manual model building. Further refinements were performed using PHENIX and COOT program. The final model of MBP-mJMJD7 contains 686 amino acid residues, 1-370 of MBP and 2–316 of mJMJD7. The structure was finally refined to 2.86 angstrom, with R/Rfree of 19.1/26.4 respectively. Model quality was checked with the PROCHECK program (https://www.ebi.ac.uk/thornton-srv/software/PROCHECK/).

**Data collection**	
Wavelength (Å)	1.0000
Space Group	$P2_1$
Resolution (Å)	62.15-2.59 (2.67-2.59)[a]
Cell parameters	
a (Å)	48.090
b (Å)	158.920
c (Å)	100.010
Observed reflections	176,892
Unique reflections ($I/\sigma(I) > 0$)	44,978
Average redundancy	3.6 (3.3)
Average $I/\sigma(I)$	5.7 (1.0)
Completeness (%)	96.4 (91.1)
R_{merge} (%)[b]	11.6 (19.7)
**Refinement**	
Resolution (Å)	62.15-2.86
Working set/test set	33,755/3,375
R_{work}/R_{free} (%)[c]	19.1/26.4
No. of protein atoms	10,880 (2 Molecules/Asym unit)
No. of water atoms	0
Root mean square deviation	
Bond lengths (Å)	0.010
Bond angles (degrees)	1.383
Ramachandran plot (%)	
Most favored regions	88.0
Allowed regions	11.7
Disallowed regions	0.1

[a] Numbers in parentheses refer to the highest resolution shell.

[b] $R_{merge}=\sum_{hkl}\sum_i|I_i(hkl) - < I(hkl)>|/ \sum_{hkl}\sum_i| I_i(hkl)|$ where $I_i(hkl)$ is the intensity of the *i*th measurement, and $< I(hkl)>$ is the mean intensity for that reflection.

[c] $R_{factor} =\sum\sum_{hkl}||Fo|-|Fc||/\sum\sum_{hkl}|Fo|$, where |Fo| and |Fc| are the observed and calculated structure factor amplitudes, respectively.

Table S3. Structure determination and refinement of mJMJD7 (PDB:4QU2).
The structure of mJMJD7 portion of MBP-mJMJD7 (PDB: 4QSZ) was used as the initial model to perform molecular replacement with PHASER. There are two Molecules in asymmetric unit. PHENIX Auto-Build was used to build model and improve the density map. COOT program was used for manual model building. Further refinements were performed using PHENIX and COOT program. The structure of mJMJD7 with a-KG was finally refined to 2.7 angstrom with R/Rfree of 20.8/25.7 respectively. The final model contains residues 1–316. Model quality was checked with the PROCHECK program (https://www.ebi.ac.uk/thornton-srv/software/PROCHECK/).

**Data collection**	
Wavelength (Å)	1.0000
Space Group	$P2_12_12_1$
Resolution (Å)	51.70-2.55 (2.65-2.55)[a]
Cell parameters	
a (Å)	53.535
b (Å)	59.516
c (Å)	199.583
Observed reflections	151,387
Unique reflections ($I/\sigma(I) > 0$)	21,313
Average redundancy	6.8 (5.6)
Average $I/\sigma(I)$	10.8 (1.8)
Completeness (%)	98.7 (92.5)
R_{merge} (%)[b]	16.6 (23.9)
**Refinement**	
Resolution (Å)	51.70-2.70
Working set/test set	17,487/1,750
R_{work}/R_{free} (%)[c]	20.80/25.70
No. of protein atoms	5,181 (2 Molecules/Asym unit)
No. of water atoms	67
Root mean square deviation	
Bond lengths (Å)	0.007
Bond angles (degrees)	1.030
Ramachandran plot (%)	
Most favored regions	87.3
Allowed regions	12.7
Disallowed regions	0

[a] Numbers in parentheses refer to the highest resolution shell.
[b] $R_{merge} = \sum_{hkl}\sum_i |I_i(hkl) - \langle I(hkl)\rangle| / \sum_{hkl}\sum_i I_i(hkl)$ where $I_i(hkl)$ is the intensity of the *i*th measurement, and $\langle I(hkl)\rangle$ is the mean intensity for that reflection.
[c] $R_{factor} = \sum\sum_{hkl}||Fo|-|Fc||/\sum\sum_{hkl}|Fo|$, where |Fo| and |Fc| are the observed and calculated structure factor amplitudes, respectively.

Table S4. Structure determination and refinement of hJMJD5 in complex with Symmetric dimethyl-Arginine at 2.2 angstrom (PDB: 6AX3) and PROCHECK (https://www.ebi.ac.uk/thornton-srv/software/PROCHECK/).

**Data collection**	
Wavelength (Å)	0.9795
Space Group	$P2_12_12_1$
Resolution (Å)	41.643-2.25 (2.33-2.25)[a]
Cell parameters	
a (Å)	49.456
b (Å)	64.706
c (Å)	77.155
Observed reflections	63,605 (14,315)
Unique reflections ($I/\sigma(I) > 0$)	11,947 (1,052)
Average redundancy	2.6 (2.3)
Average $I/\sigma(I)$	14.6 (3.1)
Completeness (%)	97.0 (95.0)
R_{merge} (%)[b]	11.0 (41.0)
**Refinement**	
Resolution (Å)	41.64-2.25
Working set/test set	11,947/1,194
R_{work}/R_{free} (%)[c]	22.03/27.03
No. of protein atoms	1,857
No. of water atoms	113
Average *B* factor of all atoms (Å^2)	
All atoms	32.35
Protein	32.16
Water	35.15
Root mean square deviation	
Bond lengths (Å)	0.009
Bond angles (degrees)	1.23
Ramachandran plot (%)	
Most favored regions	97
Allowed regions	2.1
Disallowed regions	0.43

[a] Numbers in parentheses refer to the highest resolution shell.
[b] $R_{merge} = \sum_{hkl}\sum_i |I_i(hkl) - \langle I(hkl)\rangle| / \sum_{hkl}\sum_i |I_i(hkl)|$ where $I_i(hkl)$ is the intensity of the *i*th measurement, and $\langle I(hkl)\rangle$ is the mean intensity for that reflection.
[c] $R_{factor} = \sum\sum_{hkl}||Fo|-|Fc||/\sum\sum_{hkl}|Fo|$, where |Fo| and |Fc| are the observed and calculated structure factor amplitudes, respectively.

Table S5. Structure determination and refinement of hJMJD5 in complex with monomethyl-Arginine at 2.0 angstrom (PDB:6AVS) and PROCHECK (https://www.ebi.ac.uk/thornton-srv/software/PROCHECK/)

**Data collection**	
Wavelength (Å)	0.9795
Space Group	$P2_12_12_1$
Resolution (Å)	41.93-2.00 (2.089-2.00)[a]
Cell parameters	
a (Å)	49.685
b (Å)	65.063
c (Å)	78.139
Observed reflections	111,307 (10,294)
Unique reflections ($I/\sigma(I) > 0$)	17,227 (1,652)
Average redundancy	6.5 (6.2)
Average $I/\sigma(I)$	10.64 (2.08)
Completeness (%)	99.0 (98.0)
R_{merge} (%)[b]	8.1 (47.3)
**Refinement**	
Resolution (Å)	41.93-2.00
Working set/test set	17,148/1,716
R_{work}/R_{free} (%)[c]	24.03/27.13
No. of protein atoms	1,820
No. of water atoms	89
Root mean square deviation	
Bond lengths (Å)	0.009
Bond angles (degrees)	1.271
Ramachandran plot (%)	
Most favored regions	97
Allowed regions	2.6
Disallowed regions	0

[a] Numbers in parentheses refer to the highest resolution shell.
[b] $R_{merge} = \sum_{hkl}\sum_i |I_i(hkl) - \langle I(hkl)\rangle| / \sum_{hkl}\sum_i |I_i(hkl)|$ where $I_i(hkl)$ is the intensity of the *i*th measurement, and $\langle I(hkl)\rangle$ is the mean intensity for that reflection.
[c] $R_{factor} = \sum\sum_{hkl}||Fo|-|Fc||/\sum\sum_{hkl}|Fo|$, where |Fo| and |Fc| are the observed and calculated structure factor amplitudes, respectively.

Table S6. Structure determination and refinement of hJMJD5 in complex with monomethyl-Arginine at 2.4 angstrom (PDB:5FBJ).

**Data collection**	
Wavelength (Å)	1.54178
Space Group	$P2_12_12_1$
Resolution (Å)	38.98-2.42 (2.54-2.42)[a]
Cell parameters	
a (Å)	49.539
b (Å)	65.052
c (Å)	77.950
Observed reflections	46,149
Unique reflections ($I/\sigma(I) > 0$)	10,102
Average redundancy	2.8 (2.7)
Average $I/\sigma(I)$	17.46 (4.51)
Completeness (%)	77.4 (83.1)
R_{merge} (%)[b]	8.5 (27.3)
**Refinement**	
Resolution (Å)	38.98-2.42
Working set/test set	7,775/780
R_{work}/R_{free} (%)[c]	20.42/25.66
No. of protein atoms	1,962
No. of water atoms	115
Root mean square deviation	
Bond lengths (Å)	0.005
Bond angles (degrees)	1.106
Ramachandran plot (%)	
Most favored regions	95.32
Allowed regions	4.68
Disallowed regions	0

[a] Numbers in parentheses refer to the highest resolution shell.
[b] $R_{merge}=\sum_{hkl}\sum_i|I_i(hkl) - \langle I(hkl)\rangle| / \sum_{hkl}\sum_i |I_i(hkl)|$ where $I_i(hkl)$ is the intensity of the *i*th measurement, and $\langle I(hkl)\rangle$ is the mean intensity for that reflection.
[c] $R_{factor} = \sum\sum_{hkl}||Fo|-|Fc||/\sum\sum_{hkl}|Fo|$, where |Fo| and |Fc| are the observed and calculated structure factor amplitudes, respectively.

Table S7. Peptides used in experiments
All peptides used for experiments carried in this manuscript, most of them from Anaspec: http://www.anaspec.com.

pH3R2(me2s) (AS-64631): A - R(Me2s) - TKQTARKSTGGKAPRKQLA - GGK(Biotin) - NH2.

pH4R3(me2a (AS-65278): SG - R(Me2a) - GKGGKGLGKGGAKRHRKVLRGG - K(Biotin).

pH3R2(me2a) K4(me3) (AS-65265): A - R(Me2a) - T - K(Me3) - QTARKSTGGKAPRKQLAGG - K(Biotin).

pH4(Ac5/8/12/16) (AS-65248): SGR(me2a) G - K(Ac) - GG - K(Ac) - GLG - K(Ac) - GGA - K(Ac) - RHRKVLRDNGSGS - K(Biotin).

pH4R3(me2a) (AS-65278): SG - R(Me2a) - GKGGKGLGKGGAKRHRKVLRGG - K(Biotin).

pAC-N-H4R3(me2a) (AS-64976): Ac - SG - R(me2a) - GKGGKGLGKGGAKRHRKV - GGK(biotin).

pH3 (AS-61702): ARTKQTARKSTGGKAPRKQLA - GG - K(BIOTIN) - NH2.

pH4 (AS-65097): SGRGKGGKGLGKGGAKRHRKVLRGG - K(Biotin) - NH2.

pH3K4(me2) (AS-64192): ART - K(Me2) - QTARKSTGGKAPRKQLA - GGK(Biotin) - NH2.

pH3R2(me2a) (AS-64630): A - R(Me2a) - TKQTARKSTGGKAPRKQLA - GGK(Biotin) – NH2.

pH3R8(me2a) (AS-64972): ARTKQTA - R(Me2a) - KSTGGKAPRKQLA - K(Biotin) - NH2.

Random control peptide sequence for Fluorenscence Polarization experiment: MDQLAKELTAEKR

For peptide of pH3R2(me2) +AC and pH4R3(me2) +AC are generated from pH3R2(me2) and pH4R3(me2) by adding a procedu

of artificial acetylation in vitro following protocol (Hanock and Benz. 1986. BBA. 860:699-707).
